# Predictive Antibiotic Susceptibility Testing by Next-Generation Sequencing for Periprosthetic Joint Infections: Potential and Limitations

**DOI:** 10.3390/biomedicines9080910

**Published:** 2021-07-29

**Authors:** Lukas Lüftinger, Ines Ferreira, Bernhard J. H. Frank, Stephan Beisken, Johannes Weinberger, Arndt von Haeseler, Thomas Rattei, Jochen G. Hofstaetter, Andreas E. Posch, Arne Materna

**Affiliations:** 1 Ares Genetics GmbH, Karl-Farkas-Gasse 18, 1030 Vienna, Austria; lukas.lueftinger@ares-genetics.com (L.L.); ines.ferreira@ares-genetics.com (I.F.); stephan.beisken@ares-genetics.com (S.B.); johannes.weinberger@ares-genetics.com (J.W.); andreas.posch@ares-genetics.com (A.E.P.); 2 Division of Computational Systems Biology, Department of Microbiology and Ecosystem Science, University of Vienna, 1030 Vienna, Austria; thomas.rattei@univie.ac.at; 3 Center for Integrative Bioinformatics Vienna, Max Perutz Laboratories, University of Vienna, 1030 Vienna, Austria; arndt.von.haeseler@univie.ac.at; 4 Center for Integrative Bioinformatics Vienna, Max Perutz Laboratories, Medical University of Vienna, 1030 Vienna, Austria; 5 Michael Ogon Laboratory for Orthopaedic Research, Orthopaedic Hospital Vienna-Speising, 1130 Vienna, Austria; Bernhard.Frank@oss.at (B.J.H.F.); Jochen.Hofstaetter@oss.at (J.G.H.); 6 Bioinformatics and Computational Biology, Faculty of Computer Science, University of Vienna, 1090 Vienna, Austria

**Keywords:** NGS, antibiotic susceptibility testing, machine learning, periprosthetic joint infection

## Abstract

Joint replacement surgeries are one of the most frequent medical interventions globally. Infections of prosthetic joints are a major health challenge and typically require prolonged or even indefinite antibiotic treatment. As multidrug-resistant pathogens continue to rise globally, novel diagnostics are critical to ensure appropriate treatment and help with prosthetic joint infections (PJI) management. To this end, recent studies have shown the potential of molecular methods such as next-generation sequencing to complement established phenotypic, culture-based methods. Together with advanced bioinformatics approaches, next-generation sequencing can provide comprehensive information on pathogen identity as well as antimicrobial susceptibility, potentially enabling rapid diagnosis and targeted therapy of PJIs. In this review, we summarize current developments in next generation sequencing based predictive antibiotic susceptibility testing and discuss potential and limitations for common PJI pathogens.

## 1. Introduction

The number of prosthetic joints implanted increases as people live longer and have greater aspirations for maintaining mobility [1]. In Germany alone, the number of, for example, total knee arthroplasties (TKA) is projected to rise from around 170,000 in 2018 to 225,000 in 2050. This 43% increase is accompanied by an 88% increase in revision TKAs mainly related to periprosthetic joint infections (PJIs) [2]. In general, PJIs occur in 1–4.6% of primary and revision arthroplasties [3,4]. Depending on pathogen virulence, PJI can manifest within the first weeks after prosthetic joint implantation or even months or years later. Treatment of PJIs usually entails prosthesis explantation, thorough debridement of the affected joint cavity tissue, and prosthesis replacement [5]. Following prosthesis replacement surgery, a multi-week antibiotic treatment regimen is applied to eradicate infectious pathogens. The surgical intervention and accompanying antibiotic treatment strategy depend primarily on pathogen identity and antimicrobial susceptibility status [6]. Fast and highly accurate microbial diagnostic methods, including species identification and antibiotic susceptibility testing (AST), are thus essential for achieving optimal patient outcomes.

With the evolution and increased accessibility of next-generation sequencing (NGS), infectious disease testing using NGS-based methods is becoming a viable option in clinical settings for a variety of applications [7,8]. NGS enables accurate species identification, the detection of genes related to antibiotic resistance, and the use of predictive, genome-derived AST. Moreover, when routinely available, NGS-based surveillance can contribute to the prevention of antimicrobial resistance [9,10]. The value of NGS methods in general has recently been recognized by the World Health Organization, who has recommended the adoption of NGS for infectious disease testing [11].

In this review, we discuss current approaches towards NGS based predictive AST, exemplified on common PJI pathogens, and the potential and limitations of sequencing-based methods for the management of PJIs.

## 2. Standard of Care Diagnostic Methods for PJI Management

Symptoms of PJI are often unspecific and potentially hard to distinguish from aseptic prosthesis failure [6]. Clinical diagnostic criteria and imaging methods designed to detect prosthesis loosening are non-invasive but are either associated with high intervention cost or have been found to show insufficient diagnostic sensitivity or specificity [5]. Joint aspiration of synovial fluid and subsequent microbiological and histopathological analysis is seen as the most valuable preoperative diagnostic method [6]. Pathogen identification is usually obtained using MALDI-TOF, which is limited by the databases available and might not identify some pathogens down to the species level [12].

Resistance status is typically determined via AST by microbiology culture using broth microdilution or disk diffusion, which are considered the standard reference methods [13]. Both methods can be automated in routine use and the obtained quantitative data interpreted using standardized guidelines, for example, published by the Clinical and Laboratory Standards Institute (CLSI) or European Committee on Antimicrobial Susceptibility Testing (EUCAST). Both methods, however, are inherently limited by their dependence on bacterial culture. The time-to-result for slow-growing pathogens may be days to weeks and the fraction of PJI cases attributable to non-culturable agents (culture-negative PJI, or CN-PJI) was found to be between 5% and 42% depending on the study [5,14]. In the absence of relevant information on pathogen identity, for example, CN-PJI is treated using broad-spectrum antimicrobials [5].

To help with the situation, PCR-based methods targeting known antimicrobial resistance (AMR) marker genes are increasingly used in diagnostic settings. While limited in their applicability by the number of resistance-related genes targeted by the PCR panel, performance for well-defined use cases has been encouraging. PCR-based techniques are independent from bacterial culture and operate directly on patient samples, decreasing turn-around times to a few hours [15,16]. Lausmann et al. (2020) tested the Unyvero ITI^®^ PCR cartridge on 97 patients with aseptic or septic, hip or knee revision surgery, and obtained an overall accuracy of 91.8% [17]. BioFire^®^ Bone and Joint Infection was evaluated by Graue et al. on 1544 patient samples and the obtained overall sensitivity was 90.2% and specificity was 99.8% [18]. Recently, MicroGenDX announced OrthoKEY^®^, that uses a combined approach of PCR and sequencing for infection diagnosis and management for periprosthetic joint infection [19].

## 3. Next-Generation Sequencing—Diagnostic Opportunities in PJI

With the falling cost of genome sequencing, NGS-based diagnostics are becoming attractive, that may be able to address limitations of current AST and PCR techniques [7]. Species identification by NGS has been reported to have similar or higher accuracy than the reference method MALDI-TOF and can be adapted to novel pathogen species [20,21,22]. NGS further enables in-depth strain typing and antimicrobial resistance surveillance. Species and strain information can narrow down viable treatment options, with the latter being a highly accurate predictor of antimicrobial resistance status in some pathogens, e.g., *Streptococcus pneumoniae* [23,24]. In contrast to PCR-based methods, NGS reveals information about a sample’s complete set of genetic regulatory and functional capabilities. An example of NGS usage in PJIs is reported by Ivy et al. (2018), where metagenomic shotgun sequencing was used for species identification and obtained 90% accordance to the species identified by culture in addition to identification of additional species not recovered by culture [25].

To shorten turn-around-time by circumventing microbial culture, NGS-based identification and predictive AST (pAST) might also be performed directly on native patient material. While direct sequencing of native patient samples may suffer from high human background compared to isolated microbial DNA, enrichment methods prior to sequencing (or computational methods after sequencing) can reduce the host background. A capture enrichment panel, for example, includes a selection of probes that target sequences such as AMR markers and the library size can be several orders of magnitude higher compared to PCR panels, allowing for more information to be captured, e.g., for pAST [26,27]. Enrichment panels can be of particular interest for slow-growing organisms or CN-PJIs and can be expanded to allow for species identification and detection of virulence factors as needed.

**Figure 1:**
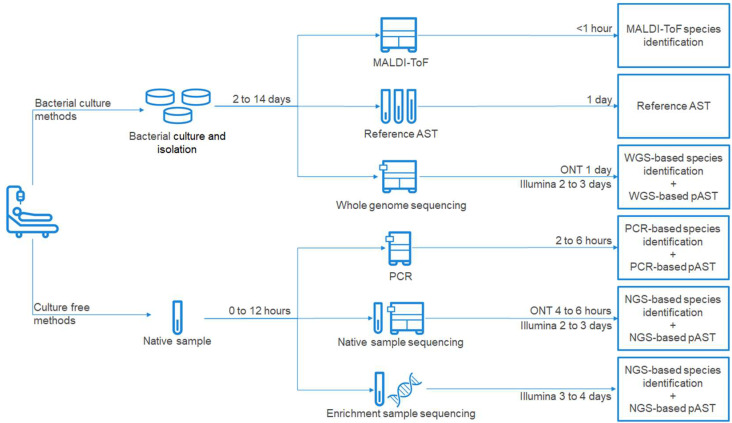
Diagram of possible routes to (predictive) AST and microbial identification. The diagram includes standard of care as well as novel methods and lists approximate times needed to obtain diagnosis.

Of note, with recent technologies such as the Oxford Nanopore Technology (ONT) platform, that sequence single DNA or RNA molecules with minimal sample preparation, additional approaches to target enrichment and direct sequencing are becoming available. ONT also allows for live result streaming for analysis and depending on the sequencer being used, only requires little bench space. Identification and predictive AST by ONT sequencing may further reduce turn-around-time [24]. Different methods and approaches that can be considered for PJI species identification and (predictive) AST are summarized in Figure 1.

## 4. Predictive Antimicrobial Susceptibility Testing

Several approaches exist for the prediction of antimicrobial susceptibility profiles from genomic data. We find that it is useful to group methods by (a) their decision algorithm and (b) their internal representation of NGS data. In this section we motivate these distinctions and introduce several key concepts of pAST as well as machine learning (ML).

### 4.1. Method Grouping by Decision Algorithm

Firstly, computational methods for pAST differ fundamentally by how resistance is determined, separating methods into rule-based or model-based. For rule-based methods, a set of rules derived by expert curation is applied to each sample, ultimately yielding the predicted resistance status. Commonly used rules pertain to the presence or absence of validated AMR resistance markers [28,29]. For example, identification of the *vanHAX* resistance cassette in an enterococcal isolate may cause calling of vancomycin resistance by a rule-based method, due to the known mechanistic link of the gene products with resistance [28].

With model-based methods, a ML model is trained on a set of samples with known resistance status to learn correlations between numerical representations of the training data and resistance status. When the trained model is then applied to samples with unknown resistance status, it returns a prediction according to the correlations learned on the training sample set. As such, an ML model trained on a training set of enterococcal isolates may report high confidence of vancomycin resistance to a sample encoding the *vanA* gene, due to the strong association of the gene with vancomycin resistance in the training set samples. Many ML algorithms have been devised, each differing in their internal strategy for optimizing prediction accuracy [30]. Several different ML algorithms have been applied to the task of predicting AST results. Common choices include ensemble methods such as XGBoost and AdaBoost, which seek to optimally combine multiple models to achieve the best performance [31,32]. Other techniques such as Support Vector Machines and Set Covering Machines attempt to find the most valuable separation criterion of samples based on the data in a single model [31,33].

Both rule-based and model-based pAST methods must be validated to ensure prediction accuracy. This is achieved by applying the method to test datasets not involved in method development and scoring predictions against reference AST measurements. ML methods are also commonly validated by splitting a single dataset into the training and test dataset, either once or repeatedly in a cross-validation scheme [30]. Clinical guidelines are used to map the quantitative output of laboratory AST assays to interpretive categories such as Resistant, Intermediate and Susceptible [34]. While most pAST methods attempt to predict interpretive categories directly, some attempt prediction of the underlying minimum inhibitory concentration (MIC), which gives insight into the strength of resistance [35]. The FDA guidance document for the performance of diagnostic AST devices can serve as a benchmark to assess performance of pAST methods. Suggested performance metrics detailed by this document are 90% categorical agreement (CA) on the interpretive category as well as at most 3% Major Error (ME) and 1.5% Very Major Error (VME), i.e., the fraction of susceptible isolates predicted as resistant and vice versa [36]. As most pAST methods predict only Resistant and Susceptible but not the Intermediate interpretive category, binary classification metrics such as accuracy, sensitivity and specificity are often provided which facilitate comparison of performance across methods. Several pAST methods do not operate on clinical interpretive categories, but on epidemiological cut-off values (ECOFFs) instead which can be defined even for pathogen/compound combinations for which no clinical guidelines are available [28,37,38]. Such methods predict wild-type/non-wild-type status of samples for a given antibiotic compound instead and likewise yield binary classification metrics.

### 4.2. Method Grouping by Internal Representation of NGS Data

A second useful distinction between pAST methods is the way the input data is structured for prediction, grouping methods into gene- and sequence-centric ones. Gene-centric methods use bioinformatics tools to annotate features like protein coding sequences, rRNA and ncRNA sequences, as well as other relevant genomic aspects in the input NGS data. In the most straight-forward case, the set of features may be restricted to established AMR marker genes or single nucleotide polymorphisms (SNPs) known to cause resistance to the compound in question. Several databases such as CARD or ResFinder curate AMR marker information and several bioinformatics tools can detect known AMR markers with high sensitivity [39,40]. The reader is referred to Anjum et al. (2017) for an in-depth review [41]. Prediction of resistance profiles is then computed from this preprocessed information using either a rule-based or model-based approach.

Conversely, sequence-centric methods utilize NGS data to make predictions without prior annotation of defined biological entities like genes and intergenic regions. Instead, input NGS data is commonly represented as the presence, absence, or abundance of so-called k-mers (DNA sequences with k base pairs) [42]. This representation does not require biological expert knowledge, and the resulting list of occurrence counts for each DNA k-mer in each sample is not easily interpretable. Thus, sequence-centric methods are usually model-based as well. By using sequence data directly, the ML model can learn the resistance status for antibiotic compounds with complex or poorly characterized mechanisms of action. Trained models can also aid in the elucidation of novel resistance mechanisms, e.g., k-mers identified as discriminators of antibiotic resistance might be mapped to known (or possibly novel) AMR marker genes and resistance-causing SNPs [35,43,44]. Importantly, sequence-centric methods also facilitate prediction from short reads directly without the need for de novo genome assembly or mapping to a reference genome and are therefore computationally efficient [28].

## 5. Advances in Predictive AST for Common PJI Pathogens

In general, pAST methods must be tailored to and evaluated on a taxon- and compound-specific basis [45]. For rule-based techniques, this entails selecting an appropriate panel of AMR markers with published relation to the resistance phenotype in question. For ML-based methods, this is achieved by training a model on a sufficiently large and diverse dataset of NGS data coupled with reference AST information. Depending on the complexity of the resistance phenotype and the taxon in question, thousands of sequenced isolates may be required to achieve optimal performance [46]. As bacterial isolate sequencing is not yet standard clinical practice, these requirements are satisfied only for a subset of bacterial taxa and antibiotic compounds. In this section we summarize current developments in the field of pAST with a focus on pathogens and antimicrobial compounds relevant to PJI diagnostics (Table 1).

### 5.1. Staphylococcus aureus and Staphylococcus *spp.*

*Staphylococcus aureus* is the leading causative agent of PJI [5]. It can efficiently colonize medical implants, forming a biofilm layer, and is implicated both in acute and chronic PJI. Antibiotic treatment options in the context of PJI include initial suppressive therapy after debridement with flucloxacillin or vancomycin (depending on methicillin resistance status) and prolonged combination therapy with the biofilm-active compound rifampin as well as fluoroquinolones or sulfonamides [6].

*S. aureus* is a promising target for predictive AST applications. As a high-impact human pathogen responsible for a range of diseases, *S. aureus* pathogenicity is well-studied and many NGS datasets relating to it are published, benefitting the development of pAST techniques [29]. The small genome of 2.8 Mbp contributes to a greater ease of classification [47]. *S. aureus* can acquire resistance mechanisms through horizontal gene transfer (HGT), leading to the emergence of highly virulent and resistant strains [48]. Nevertheless, the mere presence of canonical AMR genes is a strong determinant of resistance, with regulatory mechanisms playing a diminished role.

The relative ease of in-silico prediction of antimicrobial resistance for *S. aureus* has been demonstrated by multiple publications. In a sequence of landmark papers, Gordon et al. (2014) and Bradley et al. (2015) investigated prediction of resistance from NGS data using a curated panel of plasmid-borne resistance markers and SNPs associated with AMR [29,49]. Resistance was called if markers or SNPs associated with an antimicrobial compound were identified in assembled genomes using local sequence alignment [50]. Bradley et al. (2015) introduced the Mykrobe tool which extends this approach to raw NGS read data. Applied to an independent validation dataset, Mykrobe exhibited sensitivity and specificity of 99.1 and 99.6%, respectively, averaged over 12 antibiotic compounds (see Table 1) [29]. Davis et al. (2016) report the use of a sequence-centric ML approach that has been implemented in the automatic genome analysis pipeline of the PATRIC database [43]. Taken together, these and other studies found concordance between predicted and AST-derived susceptibility status consistently exceeding 90% [28,51].

The second major group of staphylococci known to cause PJI are coagulase-negative staphylococci (CoNS). This diverse group of human commensals and opportunistic pathogens is commonly encountered both in early-onset and late-onset PJI. Significantly less is known about virulence mechanisms and epidemiology of CoNS as compared to *S. aureus*, and few datasets suitable for machine learning are available [52]. This limits the development of both gene- and sequence-based pAST approaches. CoNS species have thus not been investigated in depth in this context. Bradley et al. (2015) report the power to discriminate *S. aureus* from CoNS species, but do not expand upon resistance prediction in the latter [29]. As resistance mechanisms are exchanged between CoNS and *S. aureus*, marker-based pAST methods developed for *S. aureus* may exhibit some level of predictiveness for CoNS [53]. CoNS have been described as the origin and reservoir of a wide array of antimicrobial resistance genes for the genus *Staphylococcus*, and broad multi-resistance as well as resistance to second line of defense drugs such as vancomycin is common in CoNS infections [54]. This shift in resistance in conjunction with the dichotomy of commensalism and pathogenicity observed in CoNS may impair predictive performance. Further research focusing on pAST for CoNS pathogens is needed

### 5.2. Enterobacterales and Non-Fermentative Gram-Negative Bacteria

Another significant group of human pathogens in the context of PJI is Gram-negative bacilli. Commonly identified causative agents include *Escherichia coli*, *Klebsiella* spp. and *Enterobacter* spp. as well as *Pseudomonas* spp. [55,56]. Ciprofloxacin is the foundation of antibiotic treatment for all susceptible Gram-negative PJI cases, with resistant cases posing a significant complication [55,57].

Despite functional similarities in resistance mechanisms, predictive performance varies substantially with pathogen species, antibiotic class and, in the case of ML-based methods, with the number of training samples. Early work by Stoesser et al. (2013) used rule-based classification by sequence alignment of assemblies to published AMR markers, followed by manual expert curation of hits to classify a small set of *E. coli* and *Klebsiella pneumoniae* isolates. This proof-of-concept study affirmed the possibility of NGS-based AST for Gram-negative bacteria, reporting excellent performance with 96% sensitivity and specificity in both species [58]. Subsequent studies investigated similar marker-gene-centric pAST techniques [59,60]. Recently, due to the availability of large NGS datasets suitable for machine learning, attention has shifted from gene-centric approaches towards sequence-centric approaches. In an extensive study of 1668 *K. pneumoniae* isolates collected from a Houston hospital system, Nguyen et al. (2018) trained gradient boosting tree ML models on DNA 10-mer representations of assembled genomes [35]. This method utilized co-resistance patterns of different antibiotic compounds to improve the final model performance but did not rely on any biological annotation of marker genes. Despite this, the authors reported concordance with resistance calls made by a phenotypic AST testing device, reaching on average 4% VME and 7% ME (see Table 1) [35]. Similarly high performance was reported by multiple other studies employing sequence-centric ML methods [44,51].

*Pseudomonas aeruginosa* is a special case among Gram-negative pathogens in the context of pAST. It is difficult to make accurate AMR predictions for relevant antibiotic compounds in this pathogen, with reported sensitivity and/or specificity metrics of around 80% for many compounds (see Table 1) [51,61]. This may be attributed to the comparatively low number of training instances available in the public domain, and to the intricate network of regulatory mechanisms governing the *P. aeruginosa* response to its environment [62]. The mutation rate of the organism, especially in hypermutator strains known to escape antibiotic treatment, further increases the genomic diversity of the species. Thus, predictive performance falls short of what is observed in species from the order Enterobacterales [51,61,63].

Much of the current treatment regimen for Gram-negative PJI hinges on resistance to ciprofloxacin. Ciprofloxacin acts via inhibition of DNA gyrase and topoisomerase, thereby killing the bacterial cell. Resistance is mediated by target modification to those proteins via amino acid substitutions in the quinolone-resistance-determining region of either protein [64]. This genotype strongly correlates with the resistance phenotype to ciprofloxacin, granting most pAST methods that can resolve the relevant SNPs excellent performance. For the Enterobacterales, performance metrics approaching and exceeding the FDA guidelines for AST devices have been reported [28,35]. The picture is more complex for *P. aeruginosa*, with multidrug efflux pumps and regulatory effects playing a significant role in the manifestation of resistance [65]. Nevertheless, sensitivity and specificity of resistance prediction of around 90% have been reported [51,61,63].

**Table 1:** Selected pAST methods targeting organisms relevant to PJI.

Organism Group	Organism	Input Data Type	Decision Algorithm Type	Data Input to Decision Algorithm	Decision Algorithm	Validation Strategy	Validation Dataset Sizes	Accuracy on Validation Datasets [# Compounds]	Year	Ref.
**Staphylococci**	*S. aureus*	Gene	Rule	AMR markers in reads or assemblies	ResFinder DB lookup	Multiple independent validation datasets	~80	96% and 97% [8]	2020	[28]
		Gene	Rule	AMR markers in reads or assemblies	Custom AMR DB lookup	Independent validation dataset	470	(Sensitivity/specificity of 99.1%/99.6%) [12]	2015	[29]
		Sequence	ML	DNA k-mer counts from assemblies	AdaBoost	10× cross-validation	11	99.5% [1]	2016	[43]
		Sequence	ML	DNA k-mer counts from reads or assemblies	Set Covering Machine	80%/20% data split	~330	95%–99% [10]	2019	[51]
**Gram-negatives**	*K. pneumoniae*	Sequence	ML	DNA k-mer counts from assemblies	XGBoost	10× cross-validation	~160	(VME/ME of 4%/7%) [21]	2018	[35]
	*E. coli*	Gene	Rule	AMR markers in reads or assemblies	ResFinder DB lookup	Multiple independent validation datasets	95, 99, 390	97%, 98% and 95% [16]	2020	[28]
		Gene	ML	gene content, isolation year, population structure	Multiple	80%/20% data split	387	91% [11]	2018	[60]
		Sequence	ML	DNA k-mer counts from reads or assemblies	Set Covering Machine	80%/20% data split	~300	80%–98% [16]	2019	[51]
	*P. aeruginosa*	Gene	ML	AMR markers, gene content, gene expression data	Support Vector Machine	80%/20% data split	~80	(Sensitivity of 81%–91%) [4]	2020	[61]
		Sequence	ML	DNA k-mer counts from reads or assemblies	Set Covering Machine	80%/20% data split	~100	73% and 95% [4]	2019	[51]
		Sequence	ML	DNA k-mer presence/absence patterns	Regression	75%/25% data split	48	88% [1]	2018	[63]
**Enterococci**	*E. faecium*	Sequence	ML	DNA k-mer counts from reads or assemblies	Set Covering Machine	80%/20% data split	27	100% [1]	2019	[51]
		Gene	Rule	AMR markers in reads or assemblies	ResFinder DB lookup	Multiple independent validation datasets	50 and 56	93% and 96% [8]	2020	[28]
	*E. faecalis*	Gene	Rule	AMR markers in reads or assemblies	ResFinder DB lookup	Independent validation dataset	50	97% [5]	2020	[28]

### 5.3. Streptococci

Pathogens from the diverse genus *Streptococcus* comprise <10% of PJI cases, but are characterized by high virulence and often point towards hematogenous origin of infection [66]. Treatment options include penicillin or ceftriaxone followed by amoxicillin or levofloxacin [6].

Streptococcal species most associated with PJI belong to the beta-hemolytic group B and G streptococci [5]. Little data is available on the effectiveness of pAST techniques for these groups. However, several published studies find promising results in the alpha-hemolytic species *Streptococcus pneumoniae*, allowing tentative extrapolation of performance in beta-hemolytic streptococci in the absence of other evidence. In a small study investigating the possibility of real-time resistance prediction during long read sequencing, Břinda et al. (2020) describe a tool which matches sequence data to a database of published *S. pneumoniae* strains, inferring resistance from database strains most closely related to the given sample. Using this technique, 91% sensitivity and 100% specificity were achieved for five clinically relevant antimicrobial compounds [24]. The extent to which this so-called genomic neighbor typing is applicable to other taxa remains unclear, as strain identity is known to be an excellent predictor of resistance status for *S. pneumoniae* specifically [67]. The obtained performance measures were broadly mirrored by figures reported by Davis et al. and Drouin et al., both using a sequence-centric ML approach [43,51].

### 5.4. Enterococci

As with streptococci, enterococcal pathogens constitute a minor fraction of PJIs (<5% of PJI cases) but are likewise identified with early-onset virulent cases [5]. Treatment choices depend largely on resistance to penicillins. Vancomycin-resistant enterococci (VRE) also exhibiting resistance to most beta-lactam antibiotics are a growing global concern, leaving only few antibiotic treatment options [68].

Overall, published performance measures of pAST applied to enterococcal pathogens are encouraging. The strong determination of vancomycin resistance by the presence of the *vanA/B* resistance gene cluster enabled both a DNA k-mer based as well as an AMR marker-based pAST approach to achieve high predictive accuracies of >98%, and performance measures were obtained for several other compounds using an AMR marker-based approach (see Table 1) [28,38,51]. A tempering factor is the generally small dataset size across all studies. More research is thus needed to confirm the applicability of these promising results for the broader population of enterococci and for other key antibiotics.

## 6. Current Limitations and Perspectives

Algorithmic advances and an increase in number and size of datasets linking genotype and resistance phenotypes have led to significant improvements in the performance of pAST methods over the last years. However, some challenges remain unsolved, some unique to PJI.

Recently published pAST methods use machine learning to correlate resistance phenotypes with sequencing data. However, ML method development may suffer from several properties inherent to biological data [69]. For example, to achieve optimal performance, most ML algorithms require significantly more training data than measured features per training example (for example presence or absence of any number of AMR marker genes). However, for biological applications, acquisition of a large and genetically diverse set of samples is often infeasible. On the other hand, measuring several features per sample is possible. The resulting so-called high-dimensional datasets are not well-suited for ML due to the increased presence of spurious correlations between samples as reviewed by Clarke et al. (2008) [69]. High dimensionality is potentiated by genome sequencing, where thousands to millions of features can be constructed from the resulting data, for example by gene calling or DNA k-mer counting. High data dimensionality is also usually accompanied by biased sampling of training data. Classically, training and validation data for ML are assumed to be sampled randomly, independently and identically from a population. In practice, this is not the case for bacterial isolates with both sequencing and AST data, where sample availability is governed by geographic location or focused on distinct outbreaks. Taken together these factors can lead to model overfitting, causing the trained model to have significantly lower accuracy when applied to independently sampled data [70]. Special care during method development must be taken to prevent this. Only a subset of ML algorithms is capable of effectively making use of high-dimensional data while minimizing overfitting [30]. Likewise, rigorous validation on independently sampled datasets is required for robust estimation of model performance in the general case [45,71]. While the increasing availability of datasets with both NGS and AST data will help in improving performance and generalizability, more research is required to establish guidelines for sampling and validation of pAST ML models that can support clinical applications.

Methods operating on data representations informed by biology can avoid some of the problems arising with sequence-centric methods. Depending on the chosen representation, data dimensionality can be significantly lower, reducing the impact of overfitting in the case of ML models. For rule-based methods, a small number of hand-curated AMR markers with published causal relation to the phenotype are employed in the decision process. This allows for increased confidence in the validity of predictions. Nevertheless, there are pitfalls even with rule-based pAST that rely on validated AMR determinants. Work on *S. aureus* shows that, in principle, marker-based methods can enable highly accurate and generalizable pAST for compounds with clearly delineated and fully understood resistance mechanisms [29]. However, a report by the European Committee on Antimicrobial Susceptibility Testing (EUCAST) in 2016 found that current public AMR databases are too sparse to support clinical pAST applications for most other pathogens [72]. This sentiment is mirrored by a recently published, comprehensive review of the state of AMR marker databases [73].

Beyond algorithmic and data representation aspects, a large fraction of PJI cases is caused by organisms that cannot readily be identified by microbiology culture to begin with. Resistance calling from culture-negative and multi-species PJI cases via clinical metagenomics is thus one of the promises of predictive AST. However, most of the methods reviewed here were developed and benchmarked on NGS data derived from cultured bacterial isolates, and transferability to metagenomics data is unclear: as most pAST methods are taxon-specific, binning of sequencing reads by pathogen taxon is necessary. Several discussed methods also require input data to be assembled into draft genomes. De novo assembly from metagenomic sequencing data however requires significantly higher read depth than assembly from isolate sequencing and introduces a host of additional biases [74]. Even methods designed to operate on raw read data have been reported to exhibit lower performance measures when applied to metagenomics datasets, thought to be attributable to large differential abundance of species in the sample (causing low abundance pathogens to be missed) as well as cross-taxon false positive hits of AMR determinants [24,29,75]. For native patient samples with low taxonomic complexity, targeted sequencing of known AMR markers is a promising intermediate step to full clinical metagenomics that warrants further research. Capture enrichment panels or adaptive sampling during ONT sequencing may enable the application of existing AMR marker-based pAST methods, which exhibit promising performance for several high impact PJI pathogens [76].

## 7. Conclusions

Compiling and contrasting pAST performances for common PJI pathogens, we find notable performance variance across species, compounds, and computational approaches. For *Staphylococcus aureus*, pAST can achieve overall performance congruent with FDA guidelines for AST testing devices, using well-understood AMR marker-based methods. For several other groups of pathogens, including the most impactful enterococci and Gram-negative bacilli, several relevant resistance phenotypes can be resolved by pAST. *Pseudomonas aeruginosa* is an outlier in the Gram-negative group, likely requiring significant additional theoretical groundwork to enable accuracies approaching FDA guidelines. Finally, for coagulase-negative staphylococci and beta-hemolytic streptococci, no conclusive statements can be made due to a lack of published data relating to the effectiveness of pAST.

With the increasing availability of NGS technology for species identification in the clinic, we expect that rule-based pAST methods targeting staphylococci may be among the first to enter clinical practice for PJI pathogens. Methods targeting most other organisms will likely require broader sampling of representative training data sets to improve and validate ML models, as well as additional study of resistance mechanisms to complement AMR marker databases.
